# Help for the Deserving

**Published:** 1876-07

**Authors:** J. H. M. Lewisburg

**Affiliations:** W. Va


*Help for the Deserving.*—The following communication is from a correspondent in West Virginia, and as his object is a meritorious one we insert it:

PROF. GORGAS—DEAR SIR : I have for some time thought that our Alumni should do our Alma Mater honor by raising an endowment fund for the education of men who are anxious to attend a Dental College, and add their names to our list of graduates. There are men who if they had the means would do honor to a diploma ; men who have a thorough classical education, and nothing is more needed in our profession now than men highly educated, yet on account of pecuniary embarrassments are not able to attend a Dental College, and they go plodding along, gaining what information they can, censured and spurned by graduates. Would it not be a noble Philanthropic enterprise to have an endowment fund, and thus aid those who are worthy, yet have not the means to attend College. There are, no doubt, talented men who would devote their lives to the profession, if they only had means to defray the expenses of a dental education, yet they will not enter our ranks to be included in the list of quacks. I cannot believe that there is an Alumnus of our proud old Alma Mater that would be so selfish as to refuse to assist in an endowment. If the desire to have highly educated men in our profession, would not urge them to do it, the love for the honor of that dear old institution that guided us as we launched our barque on life's varying tide, would do it. It would certainly not be considered unprofessional to endow a college for the benefit of poor, but worthy young men. I am young in years and scarcely started on my professional career—but I would willingly contribute my mite, hard earned though it may be. I know that there is not one in the class of '74, that would not do all in their power for an endowment fund for the Baltimore College of Dental Surgery. Students could be admitted after having passed a preliminary examination before the Faculty, on such branches as they deem necessary.

Yours respectfully,

J. H. M., Lewisburg, W. Va

